# Synergistic Modification of Steam Explosion Combined with Enzymatic Hydrolysis on Wheat Bran to Improve Dough Properties, Bread Quality, and In Vitro Digestibility

**DOI:** 10.3390/foods15091465

**Published:** 2026-04-22

**Authors:** Xiaoxuan Li, Xiaomeng Guo, Jie Yu, Zixin Zhao, Xue Tian, Wenjie Sui, Jing Meng, Tao Wu, Min Zhang

**Affiliations:** 1 State Key Laboratory of Food Nutrition and Safety, College of Food Science and Engineering, Tianjin University of Science & Technology, Tianjin 300457, China; 23842063@mail.tust.edu.cn (X.L.); 18838064356@163.com (X.G.); 23845823@mail.tust.edu.cn (J.Y.); 18832750311@163.com (Z.Z.); xuetian9701@163.com (X.T.); jing.meng@tust.edu.cn (J.M.); wutaoxx@gmail.com (T.W.); 2 Jinan Fruit Research Institute, All-China Federation of Supply & Marketing Co-Operatives, Jinan 250014, China; 3 China-Thailand Joint Research Center for Nutrition and Health Food, Tianjin Agricultural University, Tianjin 300392, China

**Keywords:** wheat bran, steam explosion, enzymatic hydrolysis, dough, bread quality

## Abstract

Wheat bran, as a major nutrient-rich agricultural by-product, is underutilized due to poor functional properties. This study investigated the synergistic effects of steam explosion (SE), enzymatic hydrolysis (EH), and SE combined with EH (SE-EH) on wheat bran to improve the dough properties, bread quality, and in vitro starch digestion. Results showed that SE destroyed the dense structure of wheat bran to form a porous surface morphology and promoted the conversion of insoluble dietary fiber (IDF) to soluble dietary fiber (SDF). This structural loosening facilitated further fiber degradation for subsequent EH and achieved the obvious improvements in hydration properties after combined treatment. For the dough system, the addition of SE-EH bran increased the water absorption, hardness, and viscosity, but reduced the development and stability time of the dough, in comparison with the control dough. These changes suggested that the modified bran altered dough hydration behavior and gluten network continuity, contributing to the increment of bread’s specific volume. The starch hydrolysis rate of bread adding SE-EH wheat bran was decreased; the slowly digestible starch (SDS) and resistant starch (RS) contents were 2.59-fold and 1.31-fold higher than the control group, respectively. Additionally, the incorporation of modified wheat bran delayed bread hardening during storage, with the SE-modified group showing the best effect. Wheat bran modification enhanced its processing functionality, providing a feasible approach for bread production to improve storage stability and nutritional quality.

## 1. Introduction

Wheat bran is a by-product of wheat flour processing and is obtained after the removal of the endosperm and germ [1]. It accounts for approximately 22% to 25% of the total wheat grain and has an annual output of about 30 million tons [2]. Wheat bran is rich in a variety of essential nutrients, including dietary fiber, arabinogalactan, B vitamins, vitamin E, and minerals [3,4]. Previous studies have shown that the dietary fiber and bioactive components present in wheat bran may contribute to reducing the risk of chronic diseases, including obesity, cardiovascular disease, and diabetes [5,6].

Owing to its role as a natural dietary fiber source, wheat bran has been widely applied in the development of baked products. However, its high lipid content, endogenous enzymes (such as lipase and lipoxygenase), and fibrous structure often lead to undesirable effects on products with high proportions of bran, including poor palatability and reduced storage stability, ultimately compromising overall product quality [7]. However, in bread making, the addition of wheat bran can interfere with gluten proteins and disrupt the continuity and stability of the gluten network, thereby adversely affecting product quality [8]. This disruption results in reduced dough elasticity and causes gas cell collapse, leading to decreased bread volume and inferior baking performance [9]. Therefore, appropriate modification of wheat bran is essential to improve the quality of bran-enriched products.

To enhance the functional properties of wheat bran, various modification strategies have been developed, including physical methods such as ultrafine grinding, extrusion, ultra-high pressure, steam explosion, thermal treatment, and high-intensity ultrasound, as well as chemical, biological (enzymatic hydrolysis and fermentation), and combined treatments. Among these approaches, chemical modification can cause severe damage to the dietary fiber structure and introduce chemical residues, raising potential food safety concerns [10]. Consequently, physical and biological methods have gained broader application due to their relatively safer and milder processing conditions. Steam explosion is regarded as a green and efficient physical modification technology. In this process, the material is exposed to high-temperature steam, and the rapid vaporization of internal moisture generates high pressure that results in structural disruption. Simultaneously, multiple physicochemical effects, including acid-like hydrolysis, thermal degradation, mechanical fragmentation, and hydrogen bond cleavage, promote the breakdown of high-molecular-weight components such as cellulose, hemicellulose, and lignin into smaller soluble dietary fiber fractions [11,12]. Studies have shown that steam explosion can significantly increase the levels of soluble dietary fiber and phenolic compounds in wheat bran, thereby improving its antioxidant activity [13,14]. It also effectively inactivates lipase and peroxidase to improve storage stability and reduce phytic acid levels [15,16]. However, the intense conditions of physical modification may compromise nutrient integrity, require substantial energy input, and demand high equipment specifications. In contrast, biological modification, particularly enzymatic treatment, offers mild reaction conditions and targeted specificity, which helps minimize nutrient loss [10]. Enzymatic modification involves cleaving glycosidic bonds within the insoluble dietary fiber matrix, reducing molecular polymerization, decreasing particle size, and loosening the texture. This process releases soluble small molecules and exposes hydrophilic functional groups, which facilitate hydrogen bonding with water and enhance the solubility of dietary fiber [17]. Although enzymatic methods enable precise control over nutritional components and improve bioavailability, their industrial application is limited by the high cost of enzymes and the complexity of optimizing enzymatic conditions [10]. Given the limitations of single-modification strategies, the combination of multiple methods has emerged as a promising trend, enabling more efficient, gentle, and low-energy modification processes [18]. By integrating physical and biological approaches, the cell walls of wheat bran can be disrupted under high temperature, pressure, and shear forces, resulting in a looser structure and greater exposure of active sites for subsequent enzymatic action. This synergistic strategy offers an efficient route for bran modification. For instance, Zhao et al. applied steam explosion combined with enzymatic hydrolysis to treat soybean residue, increasing its soluble dietary fiber content from 2.14% to 32.13%, thereby demonstrating the superiority of combined treatment. Furthermore, when the modified soybean residue was incorporated into egg tofu, the product exhibited a 2.6-fold increase in soluble dietary fiber content, along with significantly enhanced hardness, chewiness, and elastic modulus (G′), indicating improved gel properties [10].

However, most previous studies have focused on the effects of modification on the physicochemical properties of wheat bran, whereas the mechanisms involved in the combined treatment of SE and EH, along with their respective impacts on dough properties, bread quality, and in vitro digestibility, have not been fully elucidated. Therefore, this study aims to investigate the effects of SE, EH, and SE-EH modification on the composition and structure of wheat bran and the improvement of dough properties, bread quality, and in vitro starch digestion with the incorporation of modified wheat bran. For this purpose, the chemical composition, microstructure, and hydration properties of modified wheat bran were first determined. Subsequently, four types of wheat bran were used in the preparation of dough and bread. The changes in color, farinographic, and textural properties of dough induced by modified wheat bran were analyzed. Finally, the effects of modified wheat bran incorporation on the quality, in vitro digestibility, and storage stability of bread were evaluated. The results are expected to provide a theoretical basis and practical guidance for the development of high-dietary-fiber bread and the value-added utilization of wheat bran.

## 2. Materials and Methods

### 2.1. Materials

Wheat bran was donated by Shandong Fada Flour Group Co., Ltd. and stored at low temperatures to prevent mold growth. The cellulase enzyme Cellic CTec 2 was procured from Novozymes Technology Co., Ltd. (Tianjin, China).

### 2.2. Modification of Pretreatment of Wheat Bran

Raw wheat bran powder: The wheat bran was dried at 50 °C, pulverized, and passed through a 100-mesh sieve for subsequent experiments.

The steam explosion (SE) of wheat bran powder: Wheat bran was rehydrated at a solid–liquid ratio of 1:1 at room temperature for 2 h. A 500 g sample was placed in a steam explosion tank, and saturated steam was introduced to 0.8 MPa and maintained for 5 min, followed by instant pressure release. These conditions were selected based on a previous study, which demonstrated that steam explosion at 0.8 MPa for 5 min effectively disrupted the dense structure of wheat bran and promoted the dissolution of soluble dietary fiber. This condition yields the highest SDF content (9.62 g/100 g) [13].

The enzymatic hydrolysis (EH) of wheat bran powder: Dry wheat bran was used as substrate with 15% solid loading, 0.05 M citric acid was used as buffer (pH 4.8), and cellulase (5 FPU/g, filter paper activity 132 FPU/mL) was added. Hydrolysis was performed in a water bath at 50 °C with shaking at 200 r/min for 4 h. Enzyme inactivation was performed in boiling water (100 °C) for 10–15 min. After centrifugation at 3500 r/min for 20 min, the residue was collected, freeze-dried, milled, and sieved through a 100-mesh screen.

The steam explosion combined with enzymatic hydrolysis (SE-EH) of wheat bran powder: Wheat bran was first treated under the above steam explosion conditions, followed by enzymatic hydrolysis. After enzyme inactivation, centrifugation, drying, crushing, and sieving through a 100-mesh sieve, the final product was obtained.

The four final samples (raw, SE, EH, and SE-EH) were treated identically after their respective modifications. Each was dried (at 50 °C to constant weight), milled, and sieved to 100 mesh. The raw wheat bran did not undergo any of the aforementioned modification treatments, whereas the subsequent steps of drying, grinding, and sieving were identical to those applied to the modified samples. As a result, any differences observed among the four treatments can be confidently assigned to the SE, EH, and SE-EH processes, rather than to the common drying, milling, or sieving steps.

### 2.3. Determination of Physicochemical Properties of Wheat Bran

#### 2.3.1. Chemical Composition Determination

The contents of total dietary fiber (TDF), insoluble dietary fiber (IDF), and soluble dietary fiber (SDF) content were measured using the enzymatic weight method [19]; starch content was determined by the enzymatic hydrolysis method [20]; protein content was determined by the Kjeldahl method [21]; fat content was determined using Soxhlet extraction method [22]; and ash content was assessed via calcination.

#### 2.3.2. Hydration Properties Determination

The water-holding capacity (WHC), oil-holding capacity (OHC), and swelling capacity (SWC) were determined according to Twarogowska et al. [23] with minor modifications.

The method for determining WHC was as follows: 1 g of raw, SE, EH, and SE-EH wheat bran samples was weighed in a 50 mL centrifuge tube, 30 mL of distilled water was introduced to the tube, and stirred for 30 min at room temperature (20–25 °C). Then the samples were centrifuged at 2500 r/min for 20 min. The resulting supernatant was discarded, and the wet precipitate was collected and weighed. The WHC was calculated as follows:

(1) $$X=\frac{W_{2}-W_{1}}{W_{1}}$$

where *X* is the WHC of the sample (g/g), *W*_1_ is the weight of the sample (g), and *W*_2_ is the weight of the wet precipitate (g).

The method for determining OHC was as follows: 1 g of raw, SE, EH, and SE-EH wheat bran samples was weighed in a 50 mL centrifuge tube, 10 mL of soybean oil was added to the tube, vortexed (30 s), and incubated at room temperature (20–25 °C) for 1 h. Following centrifugation at 1500 r/min for 20 min, the residue was collected and weighed. OHC was computed as follows:

(2) $$X=\frac{W_{2}-W_{1}}{W_{1}}$$

where *X* is the OHC of the sample (g/g), *W*_1_ is the weight of the sample (g), and *W*_2_ is the weight of the wet (g).

The method for determining SWC was as follows: 500 mg of raw, SE, EH, and SE-EH wheat bran samples were weighed in a calibrated cylinder, and their initial volumes were recorded. Distilled water (15 mL) was added, and the mixture was thoroughly mixed and kept at room temperature for 24 h. The final volume was recorded, and the SWC was calculated as follows:

(3) $$X=\frac{V_{2}-V_{1}}{V_{1}}$$

where *X* is the SWC of the sample (g/g), *V*_1_ is the sample initial volume (mL), and *V*_2_ is the volume after standing (mL).

#### 2.3.3. Scanning Electronic Microscopy (SEM)

The wheat bran samples were observed using a JEOL JSM-3800F system (JEOL, Kyoto, Japan) to obtain SEM images at 500× magnification. The samples were dried in an oven at 60 °C. A small quantity of the sample was mounted onto the specimen stub. The samples were surface-gold-plated using a sputter-coater (Hitachi Science Systems, Kyoto, Japan). SEM analysis was conducted using a secondary electron detector with a resolution of 30 nm (at 30 kV), an accelerating voltage range of 0.3 to 30 kV, and a chamber vacuum pressure of 1.5 × 10^−3^ Pa.

### 2.4. Dough and Bread Preparation

All ingredient substitutions were performed on a dry weight (dw). Based on high-gluten wheat flour, 6 g of raw, SE, EH, and SE-EH wheat bran was used to replace part of the wheat flour (total flour: 60 g), respectively. All wheat bran powders were dried at 50 °C and passed through a 100-mesh sieve before use. The moisture contents of the high-gluten wheat flour and the raw, SE, EH, and SE-EH wheat bran were approximately 12.03%, 8.62%, 8.51%, 8.40% and 8.54%, respectively, and the whole and raw egg lipid contained about 74.01% moisture. Other ingredients were 7.5 g of sugar, 0.75 g salt, 0.80 g yeast, 2.5 g vegetable oil, and 5 g of the whole and raw egg liquid. Water addition was kept constant at 30 g of warm water (37 °C) per 60 g of flour for all dough. Based on the intrinsic water in flour, bran, and egg lipid together with the fixed 30 g water addition, the total water content of the dough was approximately 41.42–41.47 g, corresponding to an effective dough hydration of about 69.03–69.20% on a 60 g flour basis, with only minor differences among the four bran treatments. This standardized hydration level enabled direct comparison of the effects of the different bran modifications (raw, SE, EH, and SE-EH) on dough and bread properties under standardized conditions. For preparation, dry ingredients were mixed first, followed by auxiliary materials. After kneading for 5 min in a bread maker, vegetable oil was added, and kneading continued until a smooth dough with a uniform film was formed. The dough was fermented at 28 °C for 90 min, degassed, divided into 100 g pieces, and proofed at 33 °C for 30 min. Finally, baking was performed at 180 °C (upper)/200 °C (lower) for 13 min until the bread turned golden brown. One complete bread-making batch (from mixing to baking) was defined as one experimental unit. All experiments were performed with three independent biological replicates, each representing a completely separate bread-making batch (from dough mixing to final baking).

### 2.5. Determination of Color, Farinographic, and Texture of Wheat Bran Dough

#### 2.5.1. Color Measurement

A handheld colorimeter was used to determine the color of the dough. After pressing the dough into a cake, its lightness (L*), red–green value (a*), and yellow–blue value (b*) were measured. The total color difference (Δ*E*) was computed using the following equation:

(4) $$\Delta E=\sqrt{{\left(a^{*}\right)}^{2}+{\left(b^{*}\right)}^{2}+{\left(L^{*}\right)}^{2}}$$

#### 2.5.2. Texture Measurement

A texture analyzer (TA-XT Plus, Stable Micro System, Godalming, UK) was used to determine the texture of each group of wheat bran dough and bread. Dough was divided into small pieces of about 10 g each and numbered; the baked bread was cut into 2 cm × 2 cm × 2 cm cubes according to the same texture and numbered. A cylindrical probe TA10 was used for the determination with the TPA analysis mode. The test conditions were as follows: test speed of 1.00 mm/s, trigger force of 5 g, and strain of 50%. Ten replicates were performed for each measurement.

#### 2.5.3. Farinographic Properties Measurement

The impact of bran addition (10%, flour weight basis) on dough mixing behavior was characterized using a farinograph (Brabender Technologie GmbH & Co. KG, Duisburg, Germany) according to the approved American Association of Cereal Chemists (AACC) Method 54-21 [24]. The measurements were performed at a mixing bowl temperature of 30 °C and a mixing speed of 63 rpm. For each dough formulation, a sample equivalent to 300 g (14% moisture basis) was placed into the mixing bowl. Distilled water was added from a burette to achieve a dough consistency of 500 Farinograph Units (FU) as the target line. The farinographic properties were assessed by measuring key parameters including water absorption (A, %), development time (DT, min), stability time (ST, min), and degree of softening (DS, farinograph units). The results were collected using Farinograph software (Version 4.0.2, IDENT No. 72000) for operating the farinograph. The analysis of each test system was performed in triplicate.

### 2.6. Determination of Wheat Bran Bread Quality

#### 2.6.1. Basic Components Analysis

The analytical procedure for the basic composition of wheat bran bread was identical to that described in Section 2.3.1 for wheat bran.

#### 2.6.2. Specific Volume Test

The specific volume is defined as the ratio of bread volume to its mass. The baked bread was cooled to room temperature, and its mass was recorded. The bread was then placed in a 1 L graduated cylinder, which was subsequently filled with millet to the 1 L mark. The bread was removed, and the volume of millet was recorded. The difference between the total volume of the cylinder and the millet volume represented the volume of the bread.

#### 2.6.3. Internal Texture Structure Analysis

The bread samples were cut into 10 mm-thick sections using a precision slicer. Imaging of bread samples was conducted in a darkroom, using four light sources to illuminate the bread samples from different angles. A TDS126191 camera (Canon Inc., Tokyo, Japan) captured the images that were analyzed with ImageJ software (version 1.53e) to determine crumb porosity.

#### 2.6.4. Sensory Evaluation of Wheat Bran Bread

According to the national standard GB/T 20981-2021 Bread [25], 10 panelists without specific preferences were selected to conduct sensory evaluation on the finished wheat bran bread. The evaluation criteria are shown in Table 1.

### 2.7. In Vitro Starch Digestibility of Wheat Bran Bread

The in vitro starch digestibility of wheat bran bread was evaluated following a modified method of Ying et al. [26]. The bread was dried in a 60 °C lab oven for 6 h and ground into a powder. A sample (500 mg) was placed in a 50 mL centrifuge tube containing glass beads, followed by the incorporation of 25 mL of sodium acetate buffer (pH 5.2, 0.1 M). A blank tube was added with glass beads and 25 mL of HAc buffer (pH 5.2). Sample tubes and blank tubes were put into a 37 °C constant-temperature water bath shaker with agitation at 160 rpm for 10 min. The enzymatic reaction was initiated by introducing porcine pancreatic α-amylase (4 mL, 290 U/mL) and amyloglucosidase (1 mL, 60 U/mL) in a 37 °C water bath at 160 rpm. During incubation, aliquots (0.1 mL) were obtained from 0 to 180 min (0, 5, 10, 20, 30, 60, 90, 120, 150, and 180 min) and mixed with 95% ethanol (1 mL) to completely deactivate the enzymes. Following centrifugation (8000 rpm, 5 min), the supernatant was collected. After appropriate dilution, the glucose content in the supernatant following centrifugation was quantified using the 3,5-dinitrosalicylic acid (DNS) method.

The contents of rapidly digestible starch (RDS), slowly digestible starch (SDS), and resistant starch (RS) were calculated using the equations provided below:

(5) $$Hydrolysis\;rate\;\left(\%\right)=\frac{The\;content\;of\;glucose\;released\times 0.9\;}{Total\;starch\;content}\times 100\%$$

(6) $$RDS\left(\%\right)=\frac{(G_{20}-FG)\times 0.9}{TS}\times 100\%$$

(7) $$SDS(\%)=\frac{(G_{120}-G_{20})\times 0.9}{TS}\times 100\%$$

(8) $$RS\left(\%\right)=\frac{TS-RDS-SDS}{TS}\times 100\%$$

where the *FG* is the content of free reducing sugar in starch before enzymatic hydrolysis (mg), the *TS* is the total starch content in bread (mg), the *G*_20_ and *G*_120_ represent the glucose concentrations at 20 and 120 min, respectively, and the 0.9 is employed to convert these glucose values into their corresponding starch equivalents.

### 2.8. Hardness Changes in Bread During Storage

The baked bread samples were allowed to cool naturally and stored in sealed polyethylene bags at 25 °C and 50% relative humidity for further analysis. Sampling was performed on days 0, 1, 2, 3, 4, and 5 of storage. On each sampling day, a cube (2.5 × 2.5 × 2.5 cm^3^) was cut from the central part of each bread loaf. The determination method of the hardness of wheat bran bread was consistent with that of the texture of wheat bran in Section 2.5.2. Three independent bread-making batches were used for the storage experiment.

### 2.9. Statistical Analysis

Except for texture analysis, other experiments were performed with three independent experimental units per treatment (biological replicates), and three technical replicates were performed per experimental unit. For texture analysis of dough and bread, ten technical replicates were measured per experimental unit to ensure reproducibility. Statistical analysis was performed using one-way analysis of variance (ANOVA) with SPSS Statistics 24. The experimental results were presented as mean ± standard deviation, and different lowercase letters indicate significant differences among different treatment groups (significance threshold: *p* < 0.05). All figures were prepared using Origin Pro 9.0.

## 3. Results and Discussion

### 3.1. Effects of Modification Treatment on Physicochemical Properties of Wheat Bran

#### 3.1.1. Chemical Composition

**Figure 1:**
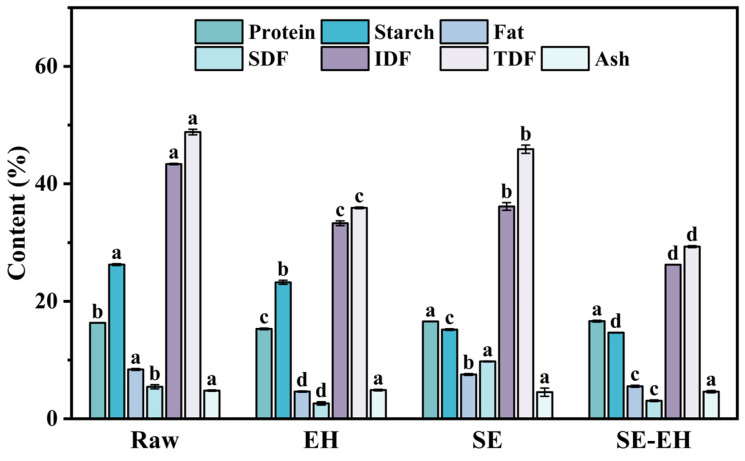
Content of wheat bran components under different modification treatments. TDF: total dietary fiber; IDF: insoluble dietary fiber; SDF: soluble dietary fiber. Different letters indicate significant differences (*p* < 0.05).

As shown in Figure 1, the effect of modification treatment on the chemical composition of wheat bran is detailed. The SDF, IDF, TDF, starch, protein, lipids, and ash of raw wheat bran are 5.45 ± 0.36%, 43.37 ± 0.13%, 48.82 ± 0.49%, 23.23 ± 0.34%, 16.34 ± 0.02%, 8.39 ± 0.16%, and 4.80 ± 0.10%, respectively. The protein and fat show no significant changes after modification treatment compared to the raw wheat bran. Compared to the starch content of raw wheat bran and SE wheat bran, the starch content decreased after EH and SE-EH treatments. This reduction can be attributed to two main factors associated with the EH process. First, during the EH process of raw and SE wheat bran, the use of cellulase disrupted the cell wall structure, thereby releasing physically entrapped starch granules, which are subsequently lost during the centrifugation step. Second, during the EH process of raw wheat bran, the hydrolysis conditions (50 °C, pH 5–6) coincidentally activated endogenous α-amylase present in wheat bran, leading to further degradation of starch into soluble sugars that were also removed during subsequent centrifugation. Compared to the raw wheat bran, the SDF content is increased by up to 1.79-fold from 5.45 ± 0.36% to 9.75 ± 0.06%, while the IDF and TDF contents decreased to 36.15 ± 0.65% and 45.90 ± 0.71% for SE samples, respectively. This phenomenon can be attributed to the high temperature-pressure conditions during SE, which partially cleave glycosidic bonds in IDF, converting insoluble fractions into soluble forms. Additionally, the hydrothermal environment promoted hemicellulose degradation and disrupted cellulose amorphous regions, further facilitating the conversion of IDF to SDF [27,28]. After EH treatment, the contents of SDF, IDF, and TDF are significantly decreased (*p* < 0.05) to 2.62 ± 0.28%, 33.29 ± 0.41%, and 35.91 ± 0.13% compared to the raw wheat bran. The cellulase can degrade the cellulose and hemicellulose in wheat bran, disrupt the cross-linking between fibers, and break macromolecular glycosidic bonds to degrade into soluble substances that are lost in the subsequent treatment [29]. After SE-EH treatment, the SDF, IDF, and TDF contents are further reduced to 3.07 ± 0.14%, 26.24 ± 0.03%, and 29.31 ± 0.17% compared to the raw wheat bran, respectively. This indicates that SE can disrupt the original porous structure of wheat bran, enhancing the accessibility of the enzyme to the substrate. Consequently, the SE-EH achieved more thorough degradation of fiber, leading to a further reduction in dietary fiber content. In conclusion, SE treatment could promote the conversion of IDF to SDF and improve the solubility of wheat bran; EH treatment could reduce various components through enzymatic degradation; thus, the SE-EH treatment could achieve the most extensive disruption of the fiber structure of wheat bran.

#### 3.1.2. Microstructure

**Figure 2:**
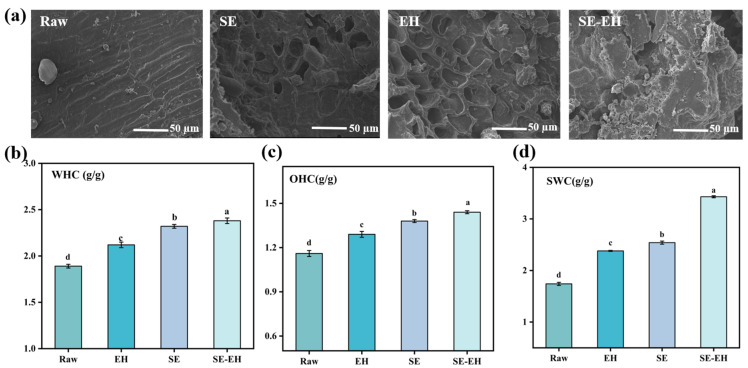
SEM images of wheat bran treated with different modification treatments (**a**); water-holding capacity (WHC), oil-holding capacity (OHC), and swelling capacity (SWC) of wheat bran with different modification treatments (**b**–**d**). Different letters indicate significant differences (*p* < 0.05).

The SEM images of the modification treatment of wheat bran are presented in Figure 2a. The raw wheat bran exhibited a compact flaky structure with a smooth and intact surface. After SE treatment, the flaky structure was significantly disrupted, resulting in the increased roughness of the surface and the formation of a clear porous structure. This morphological change could be attributed to the hydrothermal conditions during SE, which softened the wheat bran structure. During the instantaneous pressure release stage, the raw materials expanded rapidly under saturated steam pressure and high-temperature liquid water, generating immense shear forces to destroy the fiber structure and leading to fiber separation. Consequently, the porous structure of the wheat bran was damaged, and its porosity increased after SE treatment. The surface of EH-treated wheat bran became more uneven, exhibiting obvious honeycomb pores with localized agglomerations in a molten state. This occurred because cellulase penetrated the fiber interior and degraded the cell wall polysaccharides, progressively disrupting the fiber structure and increasing the structural disorder. The SE-EH treatment resulted in the most irregular surface with pronounced molten agglomerations [30]. The primary reason is that the SE treatment may disrupt the compact structure of wheat bran, forming a loose and porous morphology and increasing the specific surface area. This structural loosening facilitated the cellulase penetration and enhanced the enzyme–substrate contact during subsequent hydrolysis. The enzymatic degradation of cellulose and hemicellulose further broke down the fiber network, resulting in more fragmented structures and increased surface roughness, leading to a more pronounced molten state.

#### 3.1.3. Hydration Properties

The water-holding capacity (WHC), oil-holding capacity (OHC), and swelling capacity (SWC) of differently modified wheat bran samples are presented in Figure 2b–d. Compared to the raw wheat bran, the WHC, OHC, and SWC of modified wheat bran are increased, with the SE-EH wheat bran showing the most pronounced improvements in the three properties. The superior hydration properties of SE-EH-treated bran resulted from the synergistic effects of two modification steps. SE first disrupted the compact structure of wheat bran, making it loose and porous with increased surface area. This structural loosening enhanced enzyme accessibility to promote further degradation of IDF, exposing more hydrophilic/hydrophobic groups that enhanced WHC and OHC [31]. This extensive fragmentation of the fiber matrix by SE-EH treatment generated an expandable network that readily absorbed water and increased the volume upon hydration, thereby enhancing the SWC [30,32].

### 3.2. Effects of Modification Treatment on Color, Farinographic, and Textural Properties of Wheat Bran Dough

#### 3.2.1. Color

**Figure 3:**
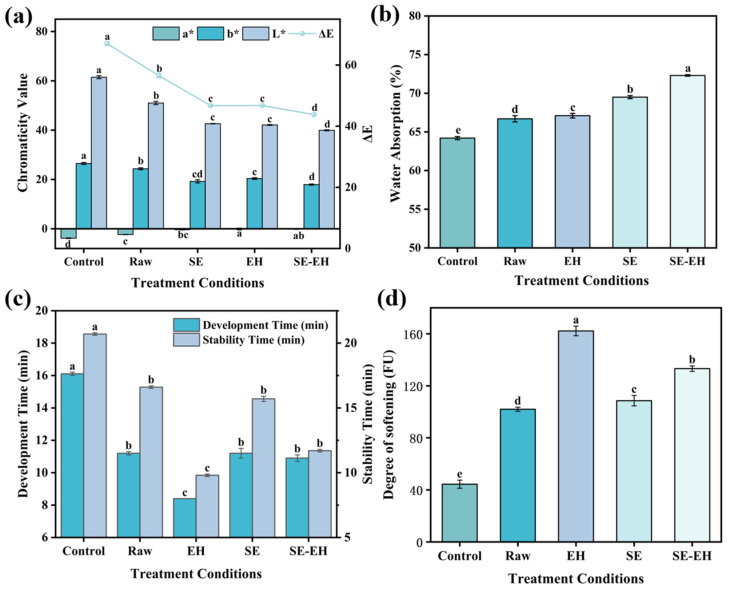
Color of dough added with wheat bran treated by different modification methods (**a**); farinographic property of dough with addition of different modified wheat bran (**b**–**d**). Different letters indicate significant differences (*p* < 0.05).

The effect of different modification methods on the color characteristics of wheat bran dough is shown in Figure 3a. L*, a*, and b* represent bright, red–green, and yellow–blue values, respectively. Compared to the control dough, the L*, b*, and ∆*E* values of dough decreased with the raw, SE, EH, and SE-EH wheat bran, while the a* values increased. The dough with SE and EH wheat bran showed similar L*, b*, and ∆*E* values, but the dough with EH wheat bran had the highest a* value. The dough with SE-EH wheat bran exhibited the lowest L*, b*, and ∆*E* values. This is because native wheat bran inherently exhibited a yellowish–white hue. During the SE progress, the Maillard reactions yielded brown melanoidin pigments, which darkened the bran powder to a yellowish–brown color [16]. During the EH progress, the soluble sugars were produced, which participated in caramelization and further Maillard reactions in the subsequent inactivation and drying process, thereby intensifying the browning of wheat bran. However, after SE-EH, the enzymatic process may dissolve some brown substances produced during SE, resulting in the lowest L*, b*, and ∆*E* values.

#### 3.2.2. Farinographic Property

The farinographic properties of dough with the addition of different types of wheat bran are shown in Figure 3b–d. Compared to the control group, the addition of different types of wheat bran significantly increased the water absorption of dough. Among all treatments, the dough with SE-EH wheat bran showed the highest water absorption rate of 72.3%. This may be attributed to the SE process, which first disrupted the dense structure of wheat bran, increasing its specific surface area and exposing hydrophilic groups. Subsequently, EH further depolymerized the fiber network, releasing hydroxyl- and carboxyl-rich groups that likely enhanced the bran’s water-retention capability. In addition, the addition of wheat bran significantly shortened dough development time and stability time while increasing the degree of softening compared to the control dough, indicating the formation of a weakened gluten network. The dough with EH wheat bran had the shortest development time and stability time, with reductions of 7.7 min and 10.9 min, respectively, and the highest degree of softening, which rose from 44.4 FU (control) to 162.2 FU compared to the control dough. The addition of wheat bran reduced the relative gluten content through a dilution effect, inhibited the hydration of gluten protein through competitive water absorption, and may have formed steric hindrance to restrict gas expansion [13,33]. The EH wheat bran has a honeycomb pore structure, which likely contributed to the fastest initial water absorption rate. The competitive water absorption of EH wheat bran resulted in insufficient hydration of gluten protein. Furthermore, the porous structure of modified wheat bran intensified the disruption of the gluten network during mixing, hindering its interaction and network formation, thereby reducing its development and stability time. Although the three types of modified wheat bran enhanced water-binding capacity, their excessive porosity and intense water competition appeared to impair gluten formation, thereby shortening dough development and stability times.

#### 3.2.3. Texture

**Figure 4:**
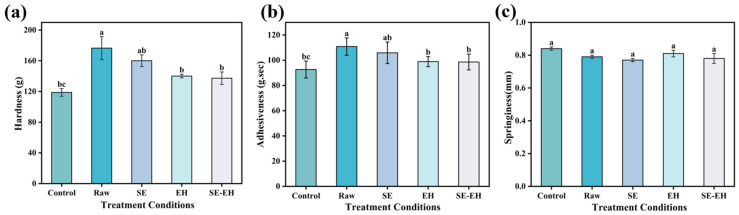
Hardness, adhesiveness, and springiness of dough with the addition of different modified wheat bran (**a**–**c**). Different letters indicate significant differences (*p* < 0.05).

The texture properties of dough with different types of wheat bran are presented in Figure 4. Compared to the control dough, the addition of wheat bran increased the hardness and adhesiveness of the dough but decreased the springiness, reflecting a tighter and less extensible gluten network. Among the treatments, the changes in the hardness and adhesiveness of dough with SE and EH wheat bran are significantly lower than those in the raw group. The hardness and adhesiveness of the SE-EH group are lower than those of the single modification group, while its springiness is intermediate between the SE and EH groups. These textural differences may be attributed to the distinct structural and compositional features of the modified brans. Wheat bran contains a high proportion of IDF, which could promote interactions with gluten proteins. Such interactions may influence the development of the gluten network, which could help explain the greater dough hardness and adhesiveness. SE wheat bran has increased SDF content and reduced IDF content, which may result in weaker interactions with gluten proteins [34]. Consequently, the hardness and adhesiveness increments in dough with SE wheat bran are significantly lower than those of the raw wheat bran group. Although the SDF content decreased in the EH wheat bran, its fiber structure became looser after enzymatic hydrolysis, possibly weakening its interactions with gluten proteins [35]. The wheat bran structure became even more porous after SE-EH treatment, allowing it to disperse more uniformly in the dough and thereby likely reducing dough hardness and adhesiveness. Structural modification of wheat bran regulated its interaction with gluten: decreased IDF content and loosened fiber morphology appear to progressively reduce excessive interactions, transforming the dough from a rigid and viscous system into a softer and more elastic matrix.

### 3.3. Effects of Modification Treatment on Quality, In Vitro Digestibility, and Storage Hardening of Wheat Bran Bread

#### 3.3.1. Basic Components

**Figure 5:**
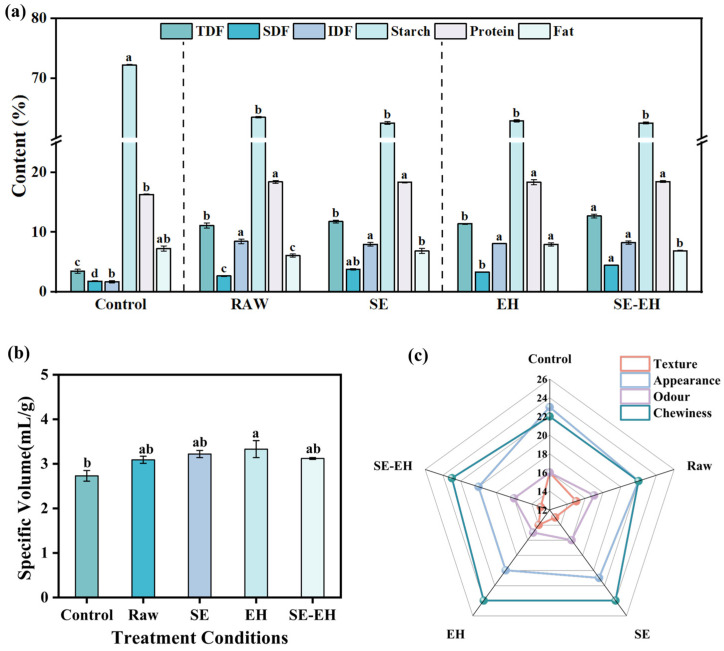
The basic components of bread with the addition of different modifications of wheat bran (**a**); the specific volume of bread (**b**); the sensory evaluation radar map of bread with the addition of different modifications of wheat bran (**c**). Different letters indicate significant differences (*p* < 0.05).

The basic components of breads with different types of wheat bran were determined, and the results are presented in Figure 5a. Compared to the control bread, breads containing wheat bran (raw, SE, EH, SE-EH) exhibited lower starch content and higher protein content. The fat content showed no significant differences between the control and breads containing bran. Among the breads made with the four-wheat bran (raw, SE, EH, SE-EH), there were no significant differences in starch, protein, and fat contents. Consequently, compared to bread with raw wheat bran, the SE, EH, and SE-EH modification treatments did not result in any significant changes in the starch, protein, and fat content of the bread. According to the “Food Nutrition Labeling Management Standard”, foods with dietary fiber content ≥6% can be labeled as “high-fiber foods”. The determination results showed that the TDF content of bread added with raw, SE-, EH-, and SE-EH-modified wheat bran exceeds 6%, indicating that bread added with wheat bran can be called a high-fiber food. The SDF content of bread added with raw, SE, EH, and SE-EH wheat bran was increased by up to 1.5-fold, 2.13-fold, 1.86-fold, and 2.51-fold compared to the control group, respectively. Among them, the bread with the addition of SE-EH wheat bran had the highest SDF content. This is because SE treatment first disrupted the dense flaky structure of raw wheat bran into a loose and porous structure, which broke the glycosidic bonds of IDF under high temperature and pressure, promoting the partial conversion of IDF to SDF and increasing the specific surface area of wheat bran. Based on this porous structure, the subsequent EH treatment allowed cellulase to fully contact the fiber substrate, further hydrolyzing cellulose and hemicellulose and releasing more soluble small-molecular fiber fragments. The SE-EH treatment showed a synergistic effect: it first disrupted the structure and then degraded the components. Consequently, the bread with the added SE-EH wheat bran has the highest SDF content, surpassing that of bread with either single modification treatment.

#### 3.3.2. Internal Texture Structure

**Figure 6:**
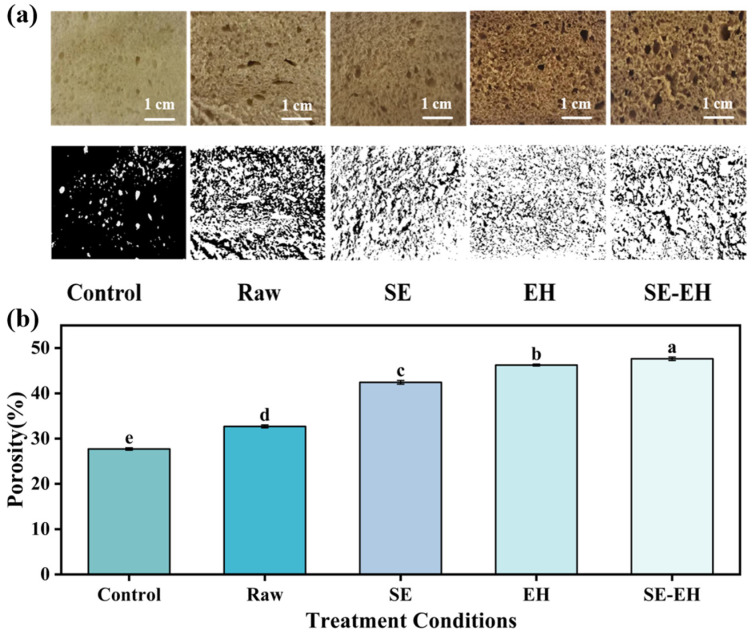
Slices (**top row**) and corresponding binary images (**bottom row**) of bread with the addition of different modifications of wheat bran (**a**); binary images obtained with ImageJ for porosity calculation (**b**). In the binary images, white pixels represent gas cells, and black pixels represent the solid bread matrix. Different letters indicate significant differences (*p* < 0.05).

The texture structure of bread is an important index for evaluating its quality. As shown in Figure 6, compared to the control sample (porosity: 27.71%), the bread incorporating raw wheat bran showed an increased porosity of 32.69%, exhibiting more and larger pores, and its texture structure was slightly rough. The bread added with SE and EH wheat bran exhibited further increased porosity of 42.43% and 46.25%, respectively, and showed the increased and enlarged pores, though the degree of pore size change was smaller than that of bread with raw wheat bran, and the texture structure was also slightly rougher than the control bread. The bread made with SE-EH wheat bran achieved the highest porosity of 47.62%, displaying the largest and most numerous pores, with the roughest texture structure. This may be attributed to the higher IDF content in raw wheat bran interacting with gluten proteins. Such interactions may affect the gluten network structure and gas retention in the dough [36], which could be associated with the larger internal pores and the rougher texture in the bread. After SE or EH treatment, the SDF content of wheat bran increased while the proportion of IDF decreased, and the wheat bran structure became loose and porous. The reduced IDF likely weakened the interaction between fibers and gluten proteins. The enhanced hydration capacity of modified bran appeared to alleviate the competitive water absorption with gluten, thus may have improved the gas retention capacity of dough, and the bread crumb formed smaller and more uniform pores compared to the raw group [37]. The SE-EH modification produced the most extensive fiber degradation, the lowest IDF content, and the most improved hydration properties in wheat bran. Though the SE-EH-modified wheat bran’s porous structure appeared to alleviate gluten–water competition, the excessively fragmented fibers may have lacked sufficient structural support for the gluten network. This structural deficiency likely led to unrestrained gas expansion and coalescence, which ultimately resulted in the largest and most numerous pores, as well as the roughest texture.

#### 3.3.3. Specific Volume

Specific volume is an essential parameter in the evaluation of bread leavening. It reflects the gas retention capacity of gluten structure to a certain extent and is used to evaluate the mass-to-volume ratio of bread. The impact of different wheat bran types on bread specific volume is presented in Figure 5b. Compared to the control group, the specific volume of bread added with raw, SE, EH, and SE-EH wheat bran increased by 13.19%, 17.95%, 21.98%, and 14.29%, respectively, with the EH group bread showing the highest specific volume. The unexpected rise in specific volume may be attributed to the fact that the WHC of wheat bran (raw and modified) influenced water distribution within the dough system and reduced structural interference with gluten, allowing for better gas retention and thus the greater bread expansion. In addition, the recipe contained sugar, oil, and whole egg, which lubricated the gluten network and softened bran particles, further mitigating the disruptive effect of bran and contributing to the observed increase in specific volume. Among treated groups, EH wheat bran exhibited a looser fiber structure and stronger interaction with gluten proteins, leading to the highest bread volume in EH bread [38].

#### 3.3.4. Sensory Analysis

The sensory evaluation radar map for breads is shown in Figure 5c. After the addition of wheat bran, the scores for internal texture and appearance are inferior to those of the control group, corresponding to the results for the bread’s internal texture structure [39]. This may be explained by the fact that adding wheat bran increased the number and size of bread pores, enhancing the rough texture and thereby affecting the internal texture and appearance. Simultaneously, the addition of wheat bran enhanced bread chewiness, with the SE and EH groups both achieving the highest chewiness score of 24. This occurred because dietary fiber could form complexes with other substances in the flour, reducing gas cells in the dough, increasing the dough cohesiveness, and resulting in better chewiness of the bread. Regarding the odor parameter, there were no significant differences in odor scores between the control bread and any of the bread containing wheat bran, indicating that the modification treatments (SE, EH, and SE-EH) did not affect the odor of the final bread products.

#### 3.3.5. In Vitro Digestibility

**Figure 7:**
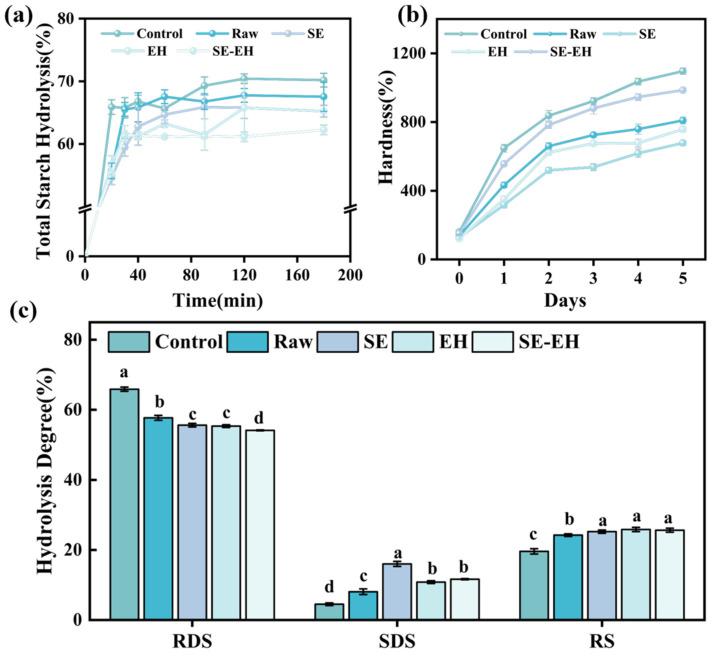
The total starch hydrolysis rate change in bread (**a**); hardness change in bread (**b**); rapidly digestible starch (RDS), slowly digestible starch (SDS), resistant starch (RS) of bread with the addition of different modified wheat bran (**c**). Different letters indicate significant differences (*p* < 0.05).

Figure 7a presents the effect of the modification process on bread starch digestibility. The starch hydrolysis of the five types of bread is the fastest during the first 30 min. At 30 min, the hydrolysis rate of bread without wheat bran is 65.79 ± 1.63%. The hydrolysis rates of bread with raw, SE, EH, and SE-EH wheat bran are 65.52 ± 1.09%, 59.46 ± 1.40%, 61.67 ± 1.24%, and 61.39 ± 0.23%, respectively. These results showed that the starch hydrolysis rate of starch in bread decreased with the addition of wheat bran. Among the four types of bread with wheat bran, the raw group had the highest hydrolysis rate, while bread with SE-EH wheat bran showed the lowest rate. Wheat bran contains abundant dietary fiber, which can physically hinder the contact between amylase and starch granules. In addition, the addition of wheat bran partially replaced flour, which had a lower starch content than the control bread, ultimately resulting in a reduced starch hydrolysis rate [40]. SE treatment caused starch gelatinization, and EH led to partial starch solubilization. Therefore, SE-EH bran had the lowest starch content, resulting in the lowest starch hydrolysis rate in bread. The SE-EH processed wheat bran shows the lowest starch content, so the bread added with SE-EH wheat bran has the lowest starch hydrolysis rate.

As shown in Figure 7c, the modification methods affect the levels of rapidly digestible starch (RDS), slowly digestible starch (SDS), and resistant starch (RS) in bread. The RDS, SDS, and RS contents of the control bread were 65.90%, 4.50%, and 19.60%, respectively. Compared to the control bread, bread samples containing differently modified wheat bran exhibited lower RDS content and higher SDS and RS contents. The bread containing SE-EH wheat bran exhibited the lowest RDS content of 54.12% and, the highest SDS content of 11.64%, and the RS content of 25.63%. These results indicated that adding the modified wheat bran could delay the rapid digestion of starch in bread. The dietary fiber of wheat bran interacted with gluten and starch, forming a physical barrier that hindered enzyme accessibility [41]. In addition, dietary fiber can interact with water to form protective complexes on the surface of starch granules, further reducing enzyme accessibility and slowing starch hydrolysis.

#### 3.3.6. Hardness Changes During Storage

The bread samples were stored at 25 °C and 50% relative humidity in sealed polyethylene bags, with hardness measured on days 0–5, and the changes in hardness of the five types of bread during storage are shown in Figure 7b. Within the same storage period, the control group exhibited the highest hardness, while bread added with SE wheat bran showed the lowest hardness. As the storage time progressed, the hardness of the four types of bread gradually increased. However, the increments in hardness for the groups with treated wheat bran are substantially reduced compared to the control group. On the 5th day of storage, the hardness of the control, raw, SE, EH, and SE-EH wheat bran breads was 6.85, 5.88, 5.32, 5.78, and 6.55 times higher than their hardness on day 1st, respectively. This indicates that the addition of wheat bran is beneficial for delaying bread staling. The dietary fiber in wheat bran exhibited excellent water-holding capacity, which hindered the moisture migration and loss during bread storage and reduced starch recrystallization [42]. Compared to other groups, SE wheat bran exhibited superior water-holding capacity due to its porous structure and higher SDF content, which significantly hindered the moisture loss during storage. Consequently, SE wheat bran delayed the rise in bread hardness to the greatest extent and improved shelf-life stability.

## 4. Conclusions

This study was conducted to address the low utilization rate of wheat bran in the food industry caused by its poor functional properties through the application of three modification methods, including SE, EH, and SE-EH treatments. The results of this study demonstrated that all three modification treatments could effectively disrupt the dense structure of wheat bran, loosen the fiber bundle, and raise the specific surface area. SE treatment first broke the glycosidic bonds of IDF through high temperature and pressure, and subsequent EH treatment further hydrolyzed cellulose and hemicellulose into soluble fragments, forming a more porous structure and promoting the conversion of IDF into SDF. Therefore, the SE-EH treatment significantly improved the hydration properties of wheat bran, enhancing its WHC, OHC, and SWC. The application of modified wheat bran has a significant impact on the processing properties of dough. Compared to the control dough, after adding SE-EH-modified wheat bran, the water absorption of dough was increased from 64.2% to 72.3%, the development time and stability time of dough were reduced, and the hardness and viscosity were increased by influencing gluten hydration and network formation. Compared to the control bread, the addition of modified wheat bran enabled the bread to be classified as a high-fiber food. Adding the EH-modified wheat bran increased the specific volume of bread by 21.98%, and improved crumb uniformity and chewiness. Adding SE-EH-modified wheat bran reduced the starch hydrolysis rate of bread from 65.79% to 61.39% and increased the contents of SDS and RS by 2.59-fold and 1.31-fold of the control values, respectively. The addition of all three modified wheat brans can effectively delay the increment of bread hardness during the storage period, ensuring the edible quality of the dietary bread during the shelf life. In conclusion, physico-chemical and biological modification of wheat bran can effectively enhance its functional properties, promote its high-value utilization in the food industry, and thus provide technical support for developing high-fiber, high-nutrition, and stable-quality bread products.

## Figures and Tables

**Table 1:** Evaluation criteria for wheat bran bread.

Indicator	Wheat Bran Bread Evaluation Criteria
Texture (20 points)	Smooth cut surface, uniform pore distribution, no flour adhesion (16–20 points) Uneven pore distribution with slight flour adhesion (10–15 points) Thick crust, rough texture, hard (0–9 points)
Appearance (30 points)	Complete and uniform shape, no adhesion or collapse (20–30 points) Cracked crust, asymmetric structure (10–19 points) Incomplete shape, severe deformation, rough surface (0–9 points)
Odor (20 points)	Characteristic wheat bran aroma (16–20 points) Faint wheat bran aroma, not obvious (10–15 points) No aroma, or bitter/astringent taste (0–9 points)
Chewiness (30 points)	Soft and delicate mouthfeel, no visible particles (20–30 points) Poor taste, bitter, with a few particles (10–19 points) Rough and sticky mouthfeel, sour and bitter (0–9 points)

## Data Availability

The original contributions presented in this study are included in the article. Further inquiries can be directed to the corresponding authors.

## Abbreviations

The following abbreviations are used in this manuscript:

SE	Steam explosion
EH	Enzymatic hydrolysis
SE-EH	Steam explosion combined with enzymatic hydrolysis
IDF	Insoluble dietary fiber
SDF	Soluble dietary fiber
TDF	Total dietary fiber
RDS	Rapidly digestible starch
SDS	Slowly digestible starch
RS	Resistant starch
WHC	Water-holding capacity
OHC	Oil-holding capacity
SWC	Swelling capacity
